# Shear bond strength of different retainer wires and bonding adhesives in consideration of the pretreatment process

**DOI:** 10.1186/1746-160X-10-51

**Published:** 2014-11-28

**Authors:** Claudia Reicheneder, Bernd Hofrichter, Andreas Faltermeier, Peter Proff, Carsten Lippold, Christian Kirschneck

**Affiliations:** Department of Orthodontics, University Medical Centre of Regensburg, Franz-Josef-Strauß-Allee 11, 93053 Regensburg, Germany; Department of Orthodontics, University of Muenster, Waldeyerstraße 30, 48149 Muenster, Germany

**Keywords:** Retainer, Wire, Adhesive, Sandblasting, Shear bond strength

## Abstract

**Introduction:**

We aimed to compare the shear bond strength (SBS) of three different retainer wires and three different bonding adhesives in consideration of the pretreatment process of enamel surface sandblasting.

**Methods:**

400 extracted bovine incisors were divided into 10 groups of 20 paired specimens each. 10 specimens of each group were pretreated by enamel sandblasting. The retainer wires Bond-A-Braid™, GAC-Wildcat®-Twistflex and everStick®ORTHO were bonded to the teeth with the adhesives Transbond™-LR, Tetric-EvoFlow™ and Stick®FLOW and then debonded measuring the SBS.

**Results:**

While sandblasting generally increased SBS for all tested combinations, the retainer wires bonded with Transbond™-LR showed the highest SBS both with and without prior sandblasting. Significantly lower SBS were found for Tetric-EvoFlow™ that were comparable to those for everStick®ORTHO.

**Conclusions:**

Pretreatment of enamel surfaces by sandblasting increased the SBS of all retainer-wires. Transbond™-LR showed the best results compared to Tetric-EvoFlow™ and everStick®ORTHO, while all combinations used provided sufficient bonding strengths for clinical use.

## Introduction

Tooth movement due to the persisting imbalance of operating forces is a major problem after orthodontic treatment [1, 2]. Stabilisation of orthodontic treatment outcomes is necessary to ensure the success of such treatments and to prevent teeth from moving back into their former position. After every orthodontic treatment, retention is essential to maintain therapy outcomes and to avoid relapse. One option is the use of retainer systems, whose positive effects have been shown before [1, 2].

Two different retainer systems, fixed and removable retainers, are used in clinical practise. Removable retainers have the disadvantages of aging, reduced wearing comfort such as impaired patient speech, and their clinical success depends on sufficient patient compliance [3]. Mainly fixed retainers guarantee the stability of anterior teeth, since they require little patient compliance. Bonded lingual retainers have become increasing popular as method of retention since the late 1970s, particularly in the mandibular incisor area [4]. Gottlieb et al. [5] reported that 81% of surveyed orthodontists use bonded lingual retainers, of which 37% use them routinely and 44% on occasion. However, retainer loss or breakage due to occlusion represents a major problem. Several authors have found that thin wires with less strands showed higher failure rates than wires with more strands or greater thickness [6, 7]. Increasing the adhesion of the retainer to the teeth has presented a major challenge.

Later developments produced wires made of fibre glass, polyethylene or Kevlar whose flexible fibres were fixated with composite. The benefits of this system include improved wearing comfort and esthetics [8, 9]. However, a clinical study by Littlewood et al. [10] showed a 3-year dropout rate of 50% for fibre-reinforced wires, whereas wires made of stainless steel had a failure rate of only 10%.

The aim of our study was to compare the SBS of two metal retainers and one fibre-reinforced retainer in combination with three different adhesive systems and to test the effect of sandblasting enamel surfaces before bonding.

## Materials and methods

**Figure 1:**
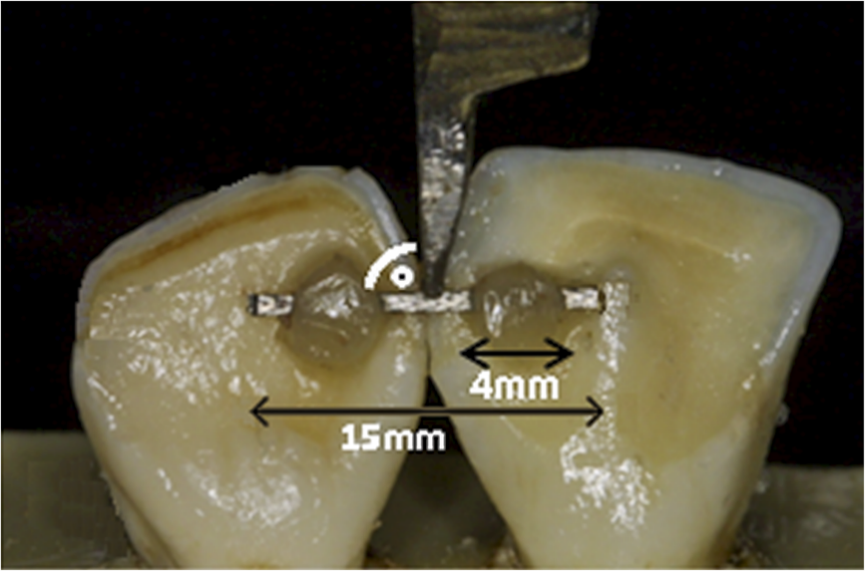
**Experimental design for testing of shear bond strength (SBS).** The force applied by the universal testing machine Instron 5965 (Instron, Pfungstadt, Germany) was directed along the occluso-apical axis of the incisors to simulate the initial bite force.

**Figure 2:**
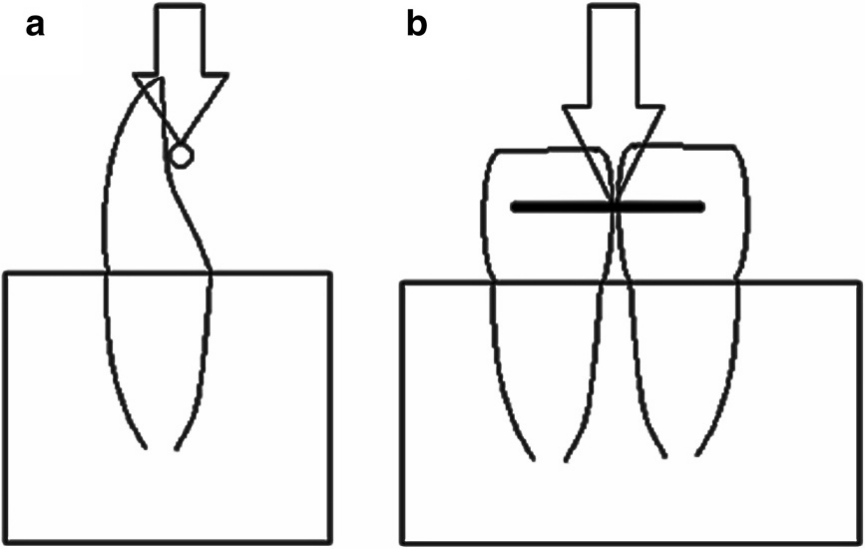
**Schematic drawing of experimental design. a** view from the side; **b** view from oral.

For this study, we used 400 freshly extracted, caries-free, and structurally intact mandibular bovine teeth. All procedures were carried out by the same operator for all test samples. The teeth were cleaned and polished for 10 seconds with pumice, disinfected with a 0.5% chloramine solution and the remaining chloramine was rinsed off to avoid chemical reactions with the adhesive. Test pairs of anatomically equal mandibular incisors were set up with approximal contact and embedded into a standardised form filled with epoxy casting resin. Surfaces were positioned in such a way that the retainer could be shorn off parallel to the crown (Figures 1 and 2). The specimens were divided into 10 groups of 20 units each. 50% of the specimens were sandblasted (KaVo PROPHYflex®, KaVo Dental GmbH, Biberach/Riss, Germany) with a fluoride-free aluminium oxide powder at an angle of 45° and a distance of 5 mm comparable to Cal-Neto et al. [11] to examine the influence of surface roughness on SBS.

Before bonding the enamel of all teeth was etched with 37% phosphoric acid (Total Etch™, Ivoclar Vivadent GmbH, Ellwangen, Germany) for 30 seconds. After the standardised appliance of a thin layer of unfilled resin (Transbond™ XT Primer, 3 M Unitek AG, Monrovia, CA, USA in the Tetric EvoFlow™ and Transbond™ LR groups and Stick Resin™, Stick Tech Ltd., Turku, Finland, in the Stick®FLOW groups) the wire was bonded with composite and light-cured with a standard curing light (Elipar™, 3 M Espe AG, Neuss, Germany) for 40 seconds. For bonding, we used three different composites: Transbond™ LR (3 M Unitek AG, Monrovia, CA, USA), a highly filled light-cured resin; Tetric EvoFlow™ (Ivoclar Vivadent GmbH, Ellwangen, Germany), a flowable light-cured nanohybrid composite and Stick®FLOW (Stick Tech Ltd., Turku, Finland), a flowable light-cured microhybrid composite. The latter was used exclusively in conjunction with the everStick® ORTHO fibre-reinforced retainer and vice versa, since they were specifically developed for combined use and Stick®FLOW is not recommended for usage in conjunction with steel retainers.Three different retainers were used in this study: Bond-A-Braid™ (Reliance Orthodontic Products Inc., Itasca, USA), an 8-times braided stainless steel retainer measuring 0.016 × 0.022 inch, GAC Wildcat® Twistflex Wire (Ortho-Care Ltd., Bradford, UK), a 3-strand twisted stainless steel retainer measuring 0.0195 inch and everStick® ORTHO (Stick Tech Ltd., Turku Finland), a fibre-reinforced retainer consisting of 1000 single unidirectional fibre-reinforced strings coated with PMMA and bis-GMA with a diameter of 0.75 mm. To standardise the bonding procedure, all retainers had a length of 15 mm. To achieve a standardised experimental procedure, we used a custom-made flexible form to position the retainer guaranteeing bonding surfaces of 4 mm in diameter located 4 mm apart from each other (Figure 1).Each specimen was stored in distilled water for 7 days before testing. For debonding we used a universal testing machine (Instron 5965, Instron, Pfungstadt, Germany) at a crosshead speed of 1 mm/min (Figure 1). The applied force was directed along the occluso-apical axis of the incisors to simulate the initial bite force. The edge of the shearing rod was positioned in the middle of the interdental segment. The load on the wire was raised until debonding occurred, and SBS was recorded in Newton (N).

The statistical analysis was performed with the software IBM SPSS Statistics 21® (IBM, Ehningen, Germany). A two-way independent factorial ANOVA was applied to compare the effect of the different retainer-adhesive-combinations and sandblasting on shear bond strength (SBS), followed-up by bootstrapped Bonferroni *post hoc* tests (1000 samples) for pairwise comparisons. Since assumptions for parametric tests were partly violated, the *post-hoc* tests were bootstrapped and nonparametric tests (Mann–Whitney U test and Kruskal-Wallis H test) used to confirm the ANOVA main effects. A p-value <0.05 was deemed significant in all cases. To assess clinical importance, effect sizes were calculated as Pearson’s correlation coefficient r with r >0.5 constituting a large, r >0.3 a medium and r >0.1 a small effect/mean difference.

## Results

**Figure 3:**
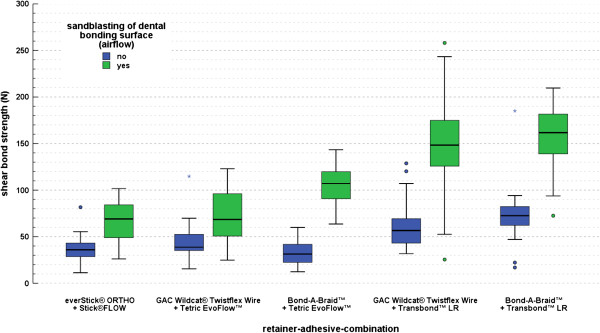
**Shear bond strengths (SBS) of the retainer-adhesive-combinations tested with and without prior sandblasting.** Boxplots show median and interquartile range while whiskers denote the data range. ° outliers (>1.5 x IQR beyond upper/lower quartile); * extreme values (>3 x IQR beyond upper/lower quartile).

The highest value for the mean shear bond strength (M = 156.33 N; SD = 36.40 N) was found for the adhesive Transbond™ LR at pretreated surfaces in combination with the retainer wire Bond-A-Braid™ (group 10) and the second highest value in combination with the retainer GAC Wildcat® Twistflex Wire (M = 146.11 N; SD = 55.03 N) (group 8) with sandblasting (Table 1, Figure 3). Tetric EvoFlow™ showed third best results at pretreated surfaces in combination with the retainer wire Bond-A-Braid™ (M = 106.55 N; SD = 21.53 N) (group 6) and the fourth best results in combination with GAC Wildcat® Twistflex Wire (M = 73.26 N; SD = 27.40 N) (group 4). The combination Bond-A-Braid™/Tetric EvoFlow™ without sandblasted surfaces (group 5) showed the lowest SBS (M = 33.55 N; SD = 13.85 N) and the combination Stick®FLOW and everStick® ORTHO (M = 37.02 N; SD = 14.79 N) without sandblasted surfaces (group 1) showed the second lowest SBS.

**Table 1:** **Shear bond strengths (SBS) of the retainer-adhesive-combinations tested with and without sandblasting**

Grp	Retainer wire	Bonding	n	pre	SBS M [N]	SBS min. [N]	SBS max. [N]	SBS SD N]
1	everStick® ORTHO	Stick®FLOW	20	no	37.02	11.22	81.67	14.79
2	everStick® ORTHO	Stick®FLOW	20	yes	65.62	26.13	101.70	22.13
3	Wildcat® Twistflex Wire	Tetric EvoFlow™	20	no	44.79	15.63	114.85	22.07
4	Wildcat® Twistflex Wire	Tetric EvoFlow™	20	yes	73.26	24.96	122.99	27.40
5	Bond-A-Braid™	Tetric EvoFlow™	20	no	33.55	12.26	59.94	13.85
6	Bond-A-Braid™	Tetric EvoFlow™	20	yes	106.55	63.60	143.52	21.53
7	Wildcat® Twistflex Wire	Transbond™ LR	20	no	63.84	31.78	128.82	29.10
8	Wildcat® Twistflex Wire	Transbond™ LR	20	yes	146.11	25.49	258.10	55.03
9	Bond-A-Braid™	Transbond™ LR	20	no	73.22	16.94	185.11	33.06
10	Bond-A-Braid™	Transbond™ LR	20	yes	156.33	72.58	209.77	36.40

M = mean; SD = standard deviation; max. = maximum; min. = minimum; Grp = test group; pre = pretreatment (enamel sandblasting).

Maximum shear bond strength values were found for the combinations Bond-A-Braid™/Transbond™ LR (209.77 N) and GAC Wildcat® Twistflex Wire/Transbond™ LR (258.10 N), both at pretreated surfaces (Table 1).

The two-way independent factorial ANOVA showed a significant and large main effect of sandblasting on the measured shear bond strength, p < 0.001, r = 0.62, i.e. mean shear bond strength was significantly higher by 217%, when the dental surfaces were sandblasted prior to bonding. This was confirmed by a Mann–Whitney-U test (Table 2).

**Table 2:** **Sandblasting significantly increased mean shear bond strength (Mann–Whitney-U-test)**

Sandblasting	n	M	SD	U	z	p	r
Yes	100	109.57	28.04	8581	8.75	<0.001	0.62
No	100	50.49	50.32				

n = number of tested specimens; M = mean; SD = standard deviation; U = test statistic Mann–Whitney-U-test; z = standardized test statistic (z-score); p = significance level; r = Pearson’s correlation coefficient (effect size).

There was a significant and large ANOVA main effect of the retainer-adhesive-combination used on the measured SBS, p < 0.001, r = 0.73, i.e. the tested combinations differed significantly in SBS. This was confirmed by a nonparametric Kruskal-Wallis-H test (n = 200, p < 0.001). The bootstrapped Bonferroni post-hoc tests showed that after surface preconditioning the combinations GAC Wildcat® Twistflex Wire/Tetric EvoFlow™ and everStick® ORTHO/Stick®FLOW did not perform significantly different when measuring SBS (p = 0.337), as was the case with the combinations GAC Wildcat® Twistflex Wire /Transbond™ LR and Bond-A-Braid™/Transbond™ LR (p = 0.488). Without surface preconditioning similar results were obtained, except for the combination Bond-A-Braid™/Tetric EvoFlow™ that did not yield significantly higher SBS values than the GAC Wildcat® Twistflex Wire/Tetric EvoFlow™ combination (Figure 3, Table 1). Sandblasting significantly affected the performance of the retainer-wire-combinations in relation to each other (ANOVA p < 0.001, r = 0.29).

The metal wire combinations bonded with Transbond LR™ showed significantly higher SBS than the wire combinations bonded with Tetric EvoFlow™, independent of the pretreatment process and the retainer wire used. However, when Tetric EvoFlow™ was used, Bond-A-Braid™ showed significantly higher SBS than GAC Wildcat® Twistflex Wire at pretreated surfaces (p = 0.001).

## Discussion

In the present study we simulated the clinical bite situation by putting a vertical thrust on the retainer. Reynolds et al. [12] and Reicheneder et al. [13] found that a vertical thrust yields the highest values of SBS compared to a tensile force in horizontal or vertical orientation. However, SBS not only depends on the direction, but also on the location of the applied force. Several authors have demonstrated that the lowest values of SBS occur when the force is applied to the interdental segment [8, 14]. Therefore we chose this most fragile segment to determine the lowest strength required for debonding.

In this study bovine teeth were used. Hobson et al. [15] recommended using incisors instead of human molars and premolars, because they found a significant difference of SBS between different tooth types. However, Nakamichi et al. [16] compared the SBS of human teeth versus bovine teeth and figured out that there was no significant difference in the results. Therefore, the use of bovine teeth seemed most suitable for our experimental purposes.

Faltermeier et al. [17] found that the SBS of two- and three-component adhesives significantly exceed that of one bottle systems. Thus in our study we only used three-component systems. However, those systems showed significant differences in bonding strength when tested with different retainer wires independent of surface pretreatment.

Zachrisson [18] and Oesterle et al. [19] enlarged the surfaces of both ends of two wires measuring 0.030 inch and 0.032 inch by sandblasting to increase the adhesion between metal and composite. We investigated the influence of sandblasting the dental bonding surface on the SBS of the retainer. The results showed that the SBS increased for all retainer-wire-combinations tested with a grand total of 217%. This indicates that sandblasting has a profound effect on clinical stability of the bonded retainer because of increased micro-retention after etching. This was confirmed by Reisner et al. [20], who examined the influence of enamel preparation on the SBS of orthodontic brackets by differentiating four groups: only sandblasted, sandblasted before etching, only acid etched and buffed with a fluted bur. They also concluded that sandblasting was no substitute for acid etching. Cal-Neto et al. [11] tested the effect of intraoral sandblasting prior to enamel etching. The bond strength increased, but the log-rank test did not show any significantly different clinical performance. In general, sandblasting not only increases the roughness of teeth, but also guarantees for a clean surface free from plaque and debris requiring only little efforts of time and material.

In our study, the Bond-A-Braid™/Transbond™ LR combination showed the highest SBS value of all combinations, followed by GAC Wildcat® Twistflex Wire/Transbond™ LR. These results indicate that the bonding strength and clinical stability of Transbond™ LR are higher than that of all other adhesive systems tested regardless of the retainer wire used.

Furthermore, the results demonstrate that the 8-times braided steel wire (Bond-A-Braid™) tolerated significantly higher SBS compared to the three-stranded wrapped steel wire (GAC Wildcat® Twistflex Wire) when used in combination with Tetric EvoFlow™, but not with Transbond™ LR. This indicates that an increased strand count of the retainer wire has generally positive effects on its clinical stability. However, since this was only true for the bonding Tetric EvoFlow™ and not for Transbond™ LR, we suppose that the influence of the bonding system on SBS is far greater than that of the retainer wire used. The SBS observed by Aldrees et al. [8] in a similar experimental set-up without prior sandblasting and using a 0.0215 inch five-stranded wrapped wire (M = 70.0 N, SD = 14.1 N) were similar to our values obtained from the woven 0.016 × 0.022 inch Bond-A-Braid™ wire without prior sandblasting (M = 73.2 N, SD = 33.1 N). This shows that other factors, such as the diameter of the wire or the amount of its windings, may be decisive for SBS.

Despite yielding excellent aesthetics, the fibre-reinforced retainers showed the lowest SBS of all tested combinations. This was also found by Foek et al. [21], who investigated the shear bond strength of steel wires compared to fibre glass wires. However, everStick® ORTHO fibre glass wire proved to be easy to handle and the observed SBS were not significantly different from those of GAC Wildcat® Twistflex Wire in combination with Tetric EvoFlow™. Furthermore, fibre glass retainers do not have to be adjusted to the dental arch prior to bonding in contrast to steel retainers. This eliminates the risk of inadvertent orthodontic force application on the retainer teeth.

Reynolds [12] determined that materials for acceptable clinical use in orthodontic treatment should be able to resist forces of 6–8 N. Waters [22] noticed that the normal range of oral forces is 3–18 N. In our study the SBS of all tested retainer systems including the fibre-reinforced systems exceeded these values and should therefore show clinically sufficient shear bond strengths.

## Conclusions

- A significant increase of the SBS could be achieved by enamel sandblasting.
- Transbond™ LR showed the best results of all the tested adhesives.
- Bond-A-Braid™ showed higher SBS and thus a lower failure rate in comparison to all other retainers tested.
- All wire/composite combinations tested provided sufficient bonding strengths for clinical use.
